# Imaging of the medial collateral ligament of the knee: a systematic review

**DOI:** 10.1007/s00402-021-04200-8

**Published:** 2021-10-10

**Authors:** P. Meyer, A. Reiter, R. Akoto, J. Steadman, G. Pagenstert, K. H. Frosch, M. Krause

**Affiliations:** 1 Department of Trauma and Orthopaedic Surgery, University Medical Center Hamburg-Eppendorf, Martinistraße 52, 20246 Hamburg, Germany; 2 Department of Trauma Surgery, Orthopaedics and Sports Traumatology, BG Hospital Hamburg, Hamburg, Germany; 3 Department of Orthopaedics, University of Utah, Salt Lake City, USA; 4 Department of Clinical Research, University of Basel, Basel, Switzerland; 5 Clarahof Clinic of Orthopaedic Surgery, Merian-Iselin-Hospital Swiss Olympic Medical Center, Basel, Switzerland; 6 Knee Institute Basel, Basel, Switzerland

**Keywords:** Knee, Medial collateral ligament, Systematic review, Ultrasonography, Magnetic resonance imaging, Stress radiography

## Abstract

**Introduction:**

The primary aim of this investigation was to systematically review relevant literature of various imaging modalities (magnetic resonance imaging (MRI), stress radiography and ultrasonography) in the assessment of patients with a medial collateral ligament (MCL) injury.

**Materials and methods:**

A systematic literature review of articles indexed in PubMed and Cochrane library was performed. Original research reporting data associated with medial gapping, surgical, and clinical findings associated with MCL injuries were considered for inclusion. The methodological quality of each inclusion was also assessed using a verified tool.

**Results:**

Twenty-three imaging studies (magnetic resonance imaging (MRI) *n* = 14; ultrasonography *n* = 6; radiography *n* = 3) were ultimately included into the review. A total of 808 injured, and 294 control, knees were assessed. Interobserver reliabilities were reported in radiographic and ultrasonographic investigations with almost perfect agreement. MRI studies demonstrated agreement ranging between substantial to almost perfect. Intraobserver reliability was only reported in radiographic studies pertinent to medial gapping and was found to be almost perfect. Correlation of MRI with clinical findings was moderate to strong (65–92%). Additionally, MRI imaging was more sensitive in the detection of MCL lesions when compared to clinical examination. However, when compared to surgical findings, MRI underestimated the grade of instability in up to 21% of cases. Furthermore, MRI showed relatively inferior performance in the identification of the exact MCL-lesion location when compared to surgical findings. Interestingly, preoperative clinical examination was slightly inferior to stress radiography in the detection of MCL lesions. However, clinical testing under general anaesthesia performed similar to stress radiography. The methodological quality analysis showed a low risk of bias regarding patient selection and index testing in each imaging modality.

**Conclusion:**

MRI can reliably diagnose an MCL lesion but demonstrates limitations in its ability to predict the specific lesion location or grade of MCL instability. Ultrasonography is a widely available, radiation free modality, but is rarely used in clinical practice for detecting MCL lesions and clinical or surgical correlates are scarce. Stress radiography findings correlate with surgical findings but clinical correlations are missing in the literature.

**Level of evidence:**

IV.

## Introduction

Injuries to the knee are associated with injury of the medial aspect of the knee in 40%, making the medial collateral ligament (MCL) complex, the most commonly injured structure of the knee. [1–4]. The MCL complex consists of three individual structures: the superficial (sMCL) and deep MCL (dMCL), as well as the posterior oblique ligament (POL). All of these structures have distinct functions in stabilizing the knee against valgus and rotatory forces [5–8]. Due to the close anatomical relationships in the knee joint, knee injuries often lead to combined soft tissue injuries and can result in complex instability [9–11]. The understanding of which, and their treatment options, remain the focus of current research [12–15]. Isolated MCL injuries lead to pain and limitations in daily, and especially, sporting activities [3]. In cases of combined injuries, including involvement of the cruciate ligaments, persistent MCL laxity seems to be an independent risk factor for failure of anterior cruciate (ACL) ligament reconstruction [16–19]. Hence, specific detection of grade and direction of MCL instability is of paramount importance.

To evaluate MCL-complex stability, different diagnostic tools exist. Initial inspection includes a physical examination which allows subjective grading of pathologic joint space opening [20–23]. Advanced imaging is also commonly utilized in the assessment of this Injury. The standard modality remains magnetic resonance imaging (MRI); however, a persistent disadvantage is its inability to perform weight-bearing acquisitions [24]. Stress radiography has also received recent positive appraisal for its ability to quantify medial joint space opening under valgus stress in the context of MCL injuries [25, 26]. Additionally, ultrasonography (US) is a cost-efficient method of diagnosing ligamentous lesions using a dynamic, real-time imaging method. However, despite these advantages, US is not a standard modality in the assessment of MCL injuries and stress radiography or MRI are commonly acquired instead [27].

Despite the various imaging modalities available to characterize MCL trauma, there still remains some degree of uncertainty of individual instability patterns seen in these injuries with imaging prior to their operative assessment. Given the paucity of data in this area, the goal of this systematic literature review is to assess the quality and evidence of various available imaging modalities in their ability to objectively describe MCL lesions.

## Materials and methods

### Search strategy

Two major medical databases: PubMed and Cochrane library were searched from inception through January 5th, 2021. The bibliographies of articles of interest were additionally reviewed. There were no limitations on the type of journal or publication date of the article. Two different keyword searches were independently performed:

1. “medial collateral ligament” OR “medial side” OR “medial instability” AND “knee” NOT “medial patellofemoral ligament” NOT “patellofemoral” NOT “total knee”.

2. “medial collateral ligament, knee” [Mesh] OR (“collateral ligament” [Ti] AND “knee” [Ti]) NOT (“patellofemoral” [Ti] OR “total knee” [Ti])”.

Additionally, a Cochrane library search with the following search string was performed: “medial collateral ligament” OR “medial side” OR “medial instability” AND “knee” NOT “medial patellofemoral ligament” NOT “patellofemoral”.

Both keyword searches were carefully merged thereafter. The systematic literature search was performed by two orthopaedic surgeons according to the guidelines of “Preferred Reporting Items for Systematic Review and Meta-Analyses” (PRISMA). The study was prospectively registered with PROSPERO (CRD42020191848; June 19th, 2020).

### Study selection

Imaging studies were included, if they were original research studies (including cadaver studies) that assessed injuries of the MCL using conventional radiography, MRI, or ultrasonography. Exclusion criteria consisted of studies published as either case reports or review articles, studies focusing on the technology of MRI imaging, studies including less than five participants, and studies not written in English. Furthermore, studies that did not have their full text available were excluded. The study selection process was conducted independently by three reviewers (M.K, P.M., A.R.). The decision to include or exclude the study was made based on a group consensus. Any deviations from consensus were discussed and resolved as a group.

### Data extraction

The following data were extracted from each imaging study: imaging modality, measurement method, number of participants and knees included, average age of participants, sensitivity and specificity, intra- and interobserver reliability, positive predictive value (PPV) and negative predictive value (NPV) and key information regarding the important radiological and clinical findings Data extraction was performed by two reviewers (P.M. and A.R.).

### Study quality assessment

The Quality Assessment of Diagnostic Accuracy Studies 2 (QUADAS-2) tool was used to assess the methodological quality of each inclusion [28]. Each study was evaluated by two reviewers (P.M. and A.R.) for risk of bias regarding patient selection, index test, reference standard, flow (e.g., lost to follow-up), and timing (e.g., time between index test and reference standard). In addition, each study was evaluated for concerns of applicability regarding patient selection, index test, and reference standard. The QUADAS checklist shows good interrater reliability as well as an excellent internal consistency and construct validity in the evaluation of musculoskeletal conditions [29].

### Statistical analysis

Data associated with the QUADAS-2 assessment were generated (Fig. 2) [28]. Mean values, positive and negative predictive values, ranges, and percentages were calculated with Microsoft Office Excel 2019.

## Results

### Literature selection

Initially, 3462 studies were found using two different search strings in 2 different databases. After removing the duplicates, 2539 publications remained. After reading the titles and abstracts, 2356 articles were excluded. The full texts of all remaining 183 articles were read and 160 articles were excluded.

**Fig. 1:**
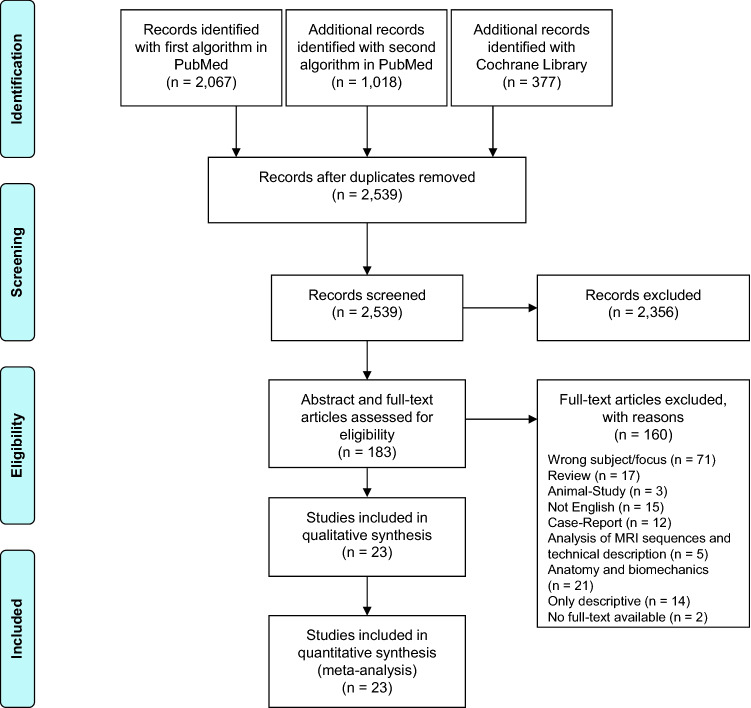
Flowchart depicting the strategy used to select relevant studies. The literature search was performed according to the guidelines of “preferred reporting items for systematic reviews and meta-analyses” (PRISMA)

In total, 23 studies were ultimately included, assessed, and underwent a quality review (Fig. 1).

### Study characteristics

Twenty-three imaging studies were included in the final analysis: 14 MRI (Table 1), 6 ultrasonography (Table 2) and 3 stress radiographic studies (Table 3). A total of 808 injured patient knees were assessed. Of this sum, 513 were from MRI studies, 119 from ultrasonography studies and 176 from stress radiographic studies. Additionally, among the included studies, 294 healthy knees were also investigated and served as a control group [25, 30–37]. The mean patient age in the majority of inclusions was under 40 years. Furthermore, a particularly young patient population was found between the included MRI studies. Overall, the mean cohort ages associated with each imaging group were comparable with ranges of 13–85, years in the MRI group, 20–63 years in the ultrasonography group and 13–67 years in the stress radiographic group, respectively. Lastly, three cadaveric studies were also included into the review, with each imaging modality represented by one study. Of these investigations, 33 total cadaver knees were evaluated.

**Table 1:** Description of studies analyzing medial collateral ligament injury with magnetic resonance imaging (MRI)

References	Patient knees/controls	Age (mean, range)	Medial joint space opening	MRI findings/main findings	Intraobserver/interobserver reliability	Sensitivity (%)/specificity (%)	PPV/NPV
Garvin et al. 1993 (RS) [41]	23/–	n.r., 16–53	n.r	Match between surgery findings and MRI: Partial rupture (low signal intensity interrupted by areas of high signal intensity): 13 of 7 cases identified Complete rupture (low signal intensity interrupted across entire width): 8 of 14 cases identified	n.r	100/n.r.^c^	n.r
Yao et al. 1994 (RS) [35]	41/22	31, 16–85	n.r	Match between clinical examinations and MRI Normal MCL: 18 of 22 cases, grade I: 7 of 13 cases, grade II: 9 of 18 cases, grade III: 7 of 10 cases	n.r	n.r	n.r
Mirowitz et al. 1994 (PS) [31]	64/10	35, 14–69	n.r	Match between clinical examinations and MRI: Grade I: 31 of 46 cases, grade II: 8 of 14 cases, grade III: 1 of 4 cases	n.r	81–100/40–95^d^	0.25–0.63/ 0.93–1.00^d^
Rasenberg et al. 1995 (PS) [32]	21/21	18, 17–50	n.r	Match between instrumented clinical examinations and MRI: Grade I: 13 of 14 cases, grade II: 5 of 5 cases, grade III: 1 of 2 cases	n.r	n.r	n.r
Schweitzer et al. 1995 (PS) [33]	76/25	26, 18–60	n.r	Correlation between MRI grade and clinical examination: Grade I (subcutaneous oedema): pain 44%, tenderness 56%, swelling 56%, instability 33% Grade II (morphologic disruption and/or internal high signal intensity and/or fluid in the MCL bursa): pain 80%, tenderness 87%, swelling 59%, instability 26% grade III (MCL discontinuity): pain 64%, tenderness 64%, swelling 50%, instability 18%	n.r./0.76–0.93	7–81/95–100^e^ (different signs)	n.r
Lundberg et al. 1996 (PS) [40]	69/–	26, 13–57	n.r	match between arthroscopy and MRI: 14 of 69 cases true positive, 41 of 69 cases true negative, 3 of 69 cases false positive, 11 of 69 cases false negative	n.r	56/93^b^	0.82/0.79^b^
De Maeseneer et al. 2001 (CS) [50]	7/6	n.r., 50–91 Control group n.r., 16–48	n.r	bursa could not be detected via MRI in cadaveric knees without contrast injection On anatomic section MCL bursa was observed in five of seven cases in the femoral and in seven of seven cases in the tibial component	n.r	n.r	n.r
Wen et al. 2007 (RS) [34]	6/12	47, n.r	n.r	Traumatic MCL-oedema: oedema deep and superficial: in six of six cases, oedema fibers: in four of six cases, bone marrow oedema: in three of six cases, medial meniscal tears: in three of six cases, medial meniscal extrusion: in one of six cases Atraumatic MCL-oedema: oedema deep and superficial: in 12 of 12 cases, oedema fibers: in 5 of 12 cases, marginal osteophytes: in 6 of 12 cases, articular cartilage thinning: in 6 of 12 cases, bone marrow oedema: in 5 of 12 cases, subchondral cysts: in 1 of 12 cases, medial meniscal tears: in 11 of 12 cases, medial meniscal extrusion: in 6 of 12 cases	n.r	n.r	n.r
Halinen et al. 2009 (PS) [39]	44/–	39, 21–64	n.r	Match between MRI and surgical treated MCL (*n* = 21): Identification of tear grade: 18 of 21 cases, identification of tear location: 11 of 21 cases	n.r	86^a^/n.r	n.r
Studler et al. 2011 (PS) [30]	10/10	35, 17–51	MCL injury: grade I: 2.3 mm, grade II: 2.9 mm (1.9–4.9 mm) control group: 1.7 mm (0.7–3.3 mm)	Grade I (*n* = 1): edema around an intact MCL Grade II (*n* = 9): partial tear of the ligament with internal high signal	n.r./ ICC: 0.89–0.94	n.r	n.r
Taketomi et al. 2014 (RS) [47]	12/–	25, 16–40	n.r	Wave sign: in all cases Identification of the ruptured end: in 9 of 12 cases Identification of entrapment: in 2 of 2 cases	n.r	n.r	n.r
Alaia et al. 2019 (RS) [48]	65/–	Only subgroups reported	n.r	Distal tibial grade III sMCL tear: in 20 of 65 cases Isolated tibial attachment tears: in 16 of 20 cases Femoral and tibial attachment tears: in 4 of 20 cases SLL: in 12 of 20 cases Borderline SLL: 6 of 20 cases	n.r	n.r	n.r
Brimmo et al. 2019 (PS) [45]	7/–	24, 16–32	n.r	SLL: redundant distal MCL fibers, displaced superficial to the pes anserinus Clinical examination: grade II in 1 of 7 cases, grade III in 6 of 7 cases	n.r	n.r	n.r
Boutin et al. 2020 (RS) [49]	51/–	28, n.r	n.r	SLL: in 20 of 51 sMCL tears Wave sign: in 18 of 20 SLL-cases and in 21 of 31 non-SLL-cases Proximal sMCL stump is located more distal and medial in cases with SLL	n.r	n.r	n.r

*CS* cadaveric study, *FS* fat-suppressed, *FSE* fast spin echo, *FSFS* fat-suppressed fluid-sensitive, *ICC* intra-class correlation coefficient, *MCL* medial collateral ligament, *NPV* negative predictive value, *n.r* not reported, *PD* proton density, *PPV* positive predictive value, *PS* prospective study, *RS* retrospective study, *SLL* Stener-like-lesion, *sMCL* superficial medial collateral ligament, *T1w* T1-weighted MRI, *T2w* T2-weighted MRI, *0.5 T* 0.5 Tesla MRI, *1.5 T* 1.5 Tesla MRI, *2D FLASH* two-dimensional fast low-angle shot, *3 T* 3 Tesla MRI

^a^Arthroscopy findings (no further definition)

^b^Arthroscopy findings: increased opening of the medial compartment

^c^Surgery findings

^d,^ ^e^Clinical examination

**Table 2:** Description of studies analyzing medial collateral ligament injury with ultrasonography

References	Patient knees/control group	Age (mean, range)	Medial joint space opening	Morphological findings	Intraobserver/interobserver reliability	Sensitivity (%)/specificity (%)	PPV/NPV
De Flaviis et al. 1988 (PS) [43]	10/–	n.r	Grade I rupture (*n* = 3): 7.0–10.0 mm Grade II rupture (*n* = 3): 9.0–18.0 mm Grade III rupture (*n* = 4): 12.0–23.0 mm	Grade I rupture: intraarticular hemorrhage Grade II rupture: inhomogenity of the ligament without a clear cut Grade III rupture: irregular hypoechoic fissure	n.r	n.r	n.r
Friedl et al. 1991 (PS) [42]	84/–	32, n.r	No rupture (*n* = 21): 2.9 ± 1.4 mm Partial rupture (*n* = 9): 5.2 ± 1.3 mm Complete rupture (*n* = 54): 6.6 ± 1.6 mm	n.r	n.r	63–87/96^a^	63–94/80
Lee et al. 1996 (PS) [36]	16/20	32, 21–52 control group: n.r., 23–28	n.r	Injured MCL (*n* = 16): “thickened, heterogenous hypoechoic lesion on the tender points” Attachment thickness: femoral 8.3 mm (6.1–12.5 mm), tibial 3.9 mm (3.7–4.1 mm) Normal MCL (*n* = 20): “homogenous hypoechotic band” Attachment thickness: femoral 4.3 mm (3.3–5.6 mm), tibial 2.3 mm (1.3–3.2 mm)	n.r	n.r	n.r
Ghosh et al. 2017 (PS) [27]	9/–	53, n.r	n.r	grade I rupture (*n* = 2): “stretching of the ligament without discontinuity of the fibers and associated edematous changes” Old rupture (*n* = 1): “Thickening of proximal MCL Normal MCL (*n* = 6): “thick hyperechoic and fibrillar structure, extending from the medial femoral condyle to the proximal tibia”	n.r	67/83^b^	67/83
Slane et al. 2017 (CS) [52]	–/8	n.r., 68–101	Unloaded: 8.7 ± 2.4 mm Loaded (10 Nm valgus): 10.7 ± 2.2 mm	n.r	n.r./ Unloaded: 0.95 Loaded: 0.93	n.r	n.r
Lutz et al. 2020 (PS) [44]	–/79	35, 20–63	Unloaded 0°: 5.7 ± 1.2 mm Loaded 0°: 7.4 ± 1.4 mm Unloaded 30°: 6.1 ± 1.1 mm Loaded 30°: 7.8 ± 1.2 mm	n.r	n.r./ ICCs: loaded and unloaded 0.89	n.r	n.r

*CS* cadaveric study, *ICC* intra-class correlation coefficient, *MCL* medial collateral ligament, *NPV* negative predictive value, *n.r* not reported, *PPV* positive predictive value, *PS* prospective study

^a^Clinical examination, examination under anaesthesia, arthroscopy, and operative findings

^b^MRI

**Table 3:** Description of studies analyzing medial collateral ligament injury with stress radiography

References	Patient knees/controls	Age (mean, range)	X-ray method	Medial joint space opening/main findings	Intraobserver/interobserver reliability	Sensitivity (%)/specificity (%)	PPV/NPV
Jacobsen et al. 1977 (PS) [25]	153/151	n.r., 13–67	Bilateral comparison with simultaneous stress at 20° flexion (9 kg)	Match between stress radiography and operative findings (not defined), medial gap difference of 2.0 mm was defined an upper limit: 63 of 89 cases true positive, 21 of 89 cases true negative, 0 of 89 cases false positive, 5 of 89 cases false negative	n.r./n.r	93/100^a^	1.00/0.81^a^
Sawant et al. 2004 (PS) [37]	23/23	33, 17–50	Bilateral comparison with simultaneous stress	MCL injury: Mean overall injuries: 16.0 mm, range: 10.0—29.0 mm Mean isolated MCL injury: 15.0 mm, range: 10.0—18.0 mm Mean combined MCL with ACL/PCL injury: 17.0 mm, range: 10.0—29.0 mm Mean control group: 8.0 mm, range: 5.0—11.0 mm	0.96/0.95	94/86^b^	0.94/0.86^b^
LaPrade et al. 2010 (CS) [26]	18/–	76, 66–86	a.p. radiographs at 0° and 20° knee FL with a fluoroscopy C-arm, clinical valgus and 10Nm two cutting sequences: 1. intact—proximal sMCL—MF—POL—distal sMCL—MT—ACL—PCL 2. intact—distal sMCL—MT—proximal sMCL—MF—POL—PCL—ACL	Sectioning of proximal sMCL: Increase of MG by 1.5 mm (at 0° knee FL) and 3.2 mm (20° knee FL) in clinical examination Sectioning of distal sMCL: Increase of MG by 2.0 mm (at 0° knee FL) and 3.1 mm (20° knee FL) in clinical examination 1. Complete meniscofemoral injury without cruciate ligament: increase of MG by 4.3 mm (at 0° knee FL) and 6.7 mm (20° knee FL) in clinical examination 2. Complete meniscotibial injury without cruciate ligament: Increase of MG by 3.6 mm (at 0° knee FL) and 5.4 mm (20° knee FL) in clinical examination	0.99/0.98	n.r./n.r	n.r./n.r

*ACL* anterior cruciate ligament, *CS* cadaveric study, *FL* flexion, MCL medial collateral ligament, *MF* meniscofemoral attachment of deep MCL, *MG* medial gap, *MT* meniscotibial attachment of deep MCL, *NPV* negative predictive value, *n.r* not reported, *PCL* posterior cruciate ligament, *POL* posterior oblique ligament, *PPV* positive predictive value, *PS* prospective study

^a^Operative findings (not defined)

^b^Arthroscopy findings (not defined)

### Quality assessment

**Fig. 2:**
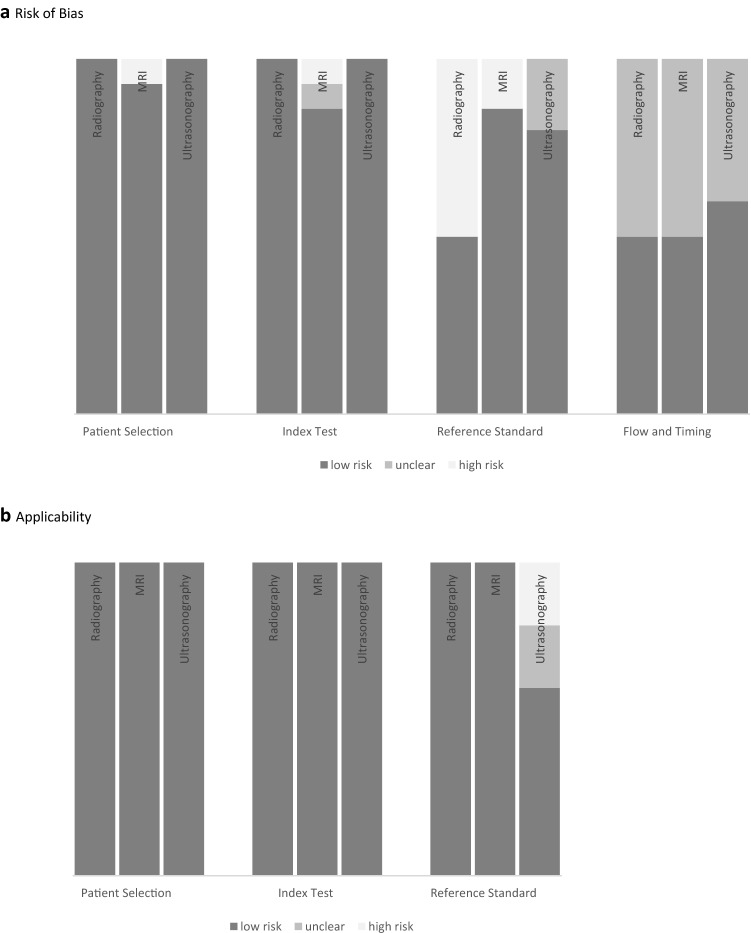
Evaluation of the Quality Assessment of Diagnostic Accuracy Studies 2 (QUADAS-2) tool to assess studies using stress radiography, magnetic resonance imaging (MRI) and ultrasonography for assessment of MCL lesions. **a** Risk of Bias. Proportion of studies with low, high or unclear risk of bias. **b** Applicability. Proportion of studies with low, high, or unclear concerns regarding applicability

The QUADAS-2 assessment demonstrated a low risk of bias regarding patient selection and index testing for all included studies. However, the risk of bias was increased for the reference standard and flow and timing. Radiographic and MRI studies showed great applicability to the reference standard. Conversely, the ultrasonography studies demonstrated a relatively lower applicability in this context (Fig. 2).

### Evaluation of imaging modalities

#### Intraobserver and interobserver reliability

Intraobserver reliability was reported in 2 of 23 studies, while interobserver reliability was reported in 6 of 23 studies. The intraobserver reliability reported within the included stress radiographic studies [26, 37] showed an almost perfect agreement (0.96–0.99) with respect to medial gapping according to Landis and Koch [38]*.* No intraobserver reliability values were reported in the MRI or ultrasonography. Concerning interobserver reliability measurement, the stress radiographic and ultrasonography studies demonstrated high agreement with almost perfect reliability (0.89–0.98) with respect to medial gapping [26, 37]. Interobserver reliability values associated with the MRI imaging studies ranged between substantial and almost perfect agreement (0.76–0.93) dependent on the specific parameter assessed in the detection of MCL lesions [33]. Lastly, the medial gapping in dynamic MRI imaging demonstrated an almost perfect interobserver agreement (0.89–0.94) [30, 33].

#### Sensitivity and specificity, positive and negative predictive values

Sensitivity was reported in eight and specificity in in 6 of 23 studies. Additionally, in one study, sensitivity and specificity were calculated from the available data. In six of these studies, furthermore PPV and NPV were reported. A cross-study reference standard did not exist and varied between clinical, arthoroscopic, and surgical findings. The greatest variance of sensitivity (7–100%) was presented in the MRI studies with respect to overall detection [39–41], classification [31] and morphological findings of an MCL lesion [33]. Sensitivity of the ultrasonography [27, 42] and stress radiographic investigations [25, 37] demonstrated a smaller variance (93–94% and 63–67%), but lower sensitivity, to detect MCL lesions through imaging. Specificity values also showed considerable variance in the MRI examinations [31, 33, 40], however, the specificity was rather moderate to high (40–100%). The ultrasonography [27, 42] and radiographic studies also saw high specificity values (83–96% and 86–100%) [25, 37].

### Medial gapping

Widening of the medial knee joint under valgus stress was evaluated in six studies, of which, two were cadaveric analyses (Table 4). Evaluation of medial gapping by ultrasonography under valgus stress showed medial gapping up to 23 mm in patients with clinically, arthroscopically, or open surgically verified complete MCL ruptures; often in combination with concomitant injuries such as lesions of the anterior cruciate ligament [42, 43].

**Table 4:** Medial joint space opening assessment of all included applicable imaging studies

References	Imaging modality	Measurement method	Medial joint space opening
De Flaviis et al. 1988 (PS) [43]	Ultrasonography	With and without valgus stress Gap width measurement: width of intraarticular space along the anterior third, the middle, and the posterior third of the internal face of the joint	Grade I rupture: 7.0–10.0 mm Grade II rupture: 9.0–18.0 mm Grade III rupture: 12.0–23.0 mm
Friedl et al. 1991 (PS) [42]	Ultrasonography	In 20° of flexion, with and without valgus stress Gap width measurement: distance between the end of the femoral condyle and tibia	No rupture: 2.9 ± 1.4 mm Partial rupture: 5.2 ± 1.3 mm Complete rupture: 6.6 ± 1.6 mm
Slane et al. 2017 (CS) [52]	Ultrasonography	Without MCL injury, in 20° of flexion, with and without valgus stress (loaded) Gap width measurement: distance between distal femur and proximal tibia relative to the skin	Unloaded: 8.7 ± 2.4 mm Loaded (10 Nm valgus): 10.7 ± 2.2 mm
Lutz et al. 2020 (PS) [44]	Ultrasonography	In 0° and 30° of flexion, unloaded and loaded valgus stress (15 daN) Gap width measurement: distance between the most medial corresponding points on the femoral condyle and tibial plateau	Unloaded 0°: 5.7 ± 1.2 mm Loaded 0° (15daN): 7.4 ± 1.4 mm Unloaded 30°: 6.1 ± 1.1 mm Loaded 30° (15daN): 7.8 ± 1.2 mm
Sawant et al. 2004 (PS) [37]	Radiography	In 10–15° of flexion, with valgus stress Gap width measurement: most medial distance between femoral condyle and corresponding medial tibial plateau	Mean only isolated MCL injury: 15 mm, range: 10–18 mm Mean combined MCL with ACL and/or PCL injury: 17 mm, range: 10–29 mm
LaPrade et al. 2010 (CS) [26]	Radiography	In 0° and 20° of flexion, with valgus stress (clinical and loaded), before and after sectioning Gap width measurement: shortest distance between the subchondral bone surface of the most distal aspect of the medial femoral condyle and the corresponding medial tibial plateau	Increase of MG in clinical examination 20° knee FL Sectioning of proximal sMCL: by 3.2 mm to 10.6 ± 1.9 mm Sectioning of distal sMCL: by 3.1 mm to 10.6 ± 1.4 mm Complete meniscofemoral injury: by 6.7 mm to 14.1 ± 2.1 mm Complete meniscotibial injury: by 5.4 mm to 12.9 ± 2.2 mm
Studler et al. 2011 (PS) [30]	dynamic MRI	Clinical examination with valgus stress Gap width measurement: distance between medial tibial margin and the cortex of the medial femoral condyles	Grade I rupture: 2.3 mm Grade II rupture: 2.9 mm (1.9–4.9 mm)

*ACL* anterior cruciate ligament, *CS* cadaveric study, *daN* dekanewton, *FL* flexion, *MCL* medial collateral ligament, *MF* meniscofemoral attachment of deep MCL, *MG* medial gap, *MT* meniscotibial attachment of deep MCL, n.r*.* not reported, *PCL* posterior cruciate ligament, *POL* posterior oblique ligament, *PS* prospective study

Stress radiography in patients with acute medial knee injuries demonstrated similar results with a medial joint space opening as large as 29 mm [37]. In comparison, the simulation of various injury patterns of the MCL, by stepwise cutting, resulted in lower medial gapping values in cases of a complete meniscofemoral or meniscotibial injury in cadaveric specimens [26]. Overall, patients with an isolated MCL lesion had a smaller maximal medial gapping of 18 mm compared to patients with a combined injury of the MCL and one or both cruciate ligaments that allowed a medial gapping as great as 29 mm [26, 37]. Comparative analyses in healthy knees under valgus stress showed that a medial gapping of 3–11 mm can occur, even in the absence of medial joint pathology [44].

### Correlation with clinical findings

Comparative clinical grades of MCL lesions were reported in 5 of 13 MRI studies (Table 1). The clinical examination focused on medial gapping and laxity grades, utilizing novel graduations defined by the respective authors [31, 32, 45], the Fetto et al. method [21, 30], or the Hughston et al. method [20, 35]. Additionally, in one inclusion, only clinical symptoms such as pain, tenderness, swelling and instability were evaluated [33]. A moderate-to-strong correlation of 65–92% between clinical findings and MRI results was reported [46]. MRI was found to demonstrate higher sensitivity in the detection of MCL lesions relative to clinical examination. Mild lesions, particularly, commonly demonstrated MRI signs such as oedema, hyperintensity, ligamentous disruption, or even detachment but proved to be clinically stable and inconspicuous under valgus stress in a physical examination [31, 35]. In three further studies, the clinical examination results were described, but no comparison was reported to MRI findings. Additionally, clinical examination findings were reported in three of the six ultrasonography studies. However, the correlation between ultrasonography and clinical findings remains unclear based on the data present in available literature. There were no studies comparing stress radiography with clinical findings.

### Correlation with surgical and arthroscopic findings

Surgical or arthroscopic findings were reported in 4 of 13 MRI studies. In three of these articles, lesion grades were compared [39–41]. MRI examination was found to underestimate the actual grade of MCL-lesion instability and failed to recommend necessary surgery in 21% of cases when compared to intraoperative findings. Furthermore, MRI performed inferior relative to intraoperative diagnosis in its ability to identify the exact MCL-lesion location. The agreement of localization between these two diagnostic methods saw only moderate values at 52% and 75% [39, 47]. Surgical findings of the MCL were also recorded in a sample of the included ultrasonography and radiographic studies, however, in most these studies, no individual comparative findings were described [37, 42, 43]. Only one radiographic study correlated their results under valgus stress in 20° flexion with operative findings. In this investigation, stress radiography of lesions acquired within 14 days of onset correctly identified MCL injuries with high accuracy (positive predictive value 100%, negative predictive value 81%) relative to operative findings. Interestingly, preoperative clinical examination under general anaesthesia of the MCL lesions was slightly inferior to stress radiography with 12 false positive cases compared to surgical findings that were verified intraoperatively as “damages to the ligaments or capsular structures” [25].

## Discussion

The primary findings of the present study demonstrate that (1) from a sparse number of available studies, interobserver reliability of stress radiography and ultrasonography in the assessment of medial gapping, and MRI in its ability to detect MCL lesions was high. (2) Medial gapping width reliably correlates with clinically and surgically verified MCL lesions. (3) Correlation between MRI lesion grading and clinical examination lesion grading is moderate to strong and MRI findings may overestimate injury severity with respect to clinical lesion stability. (4) Correlation between MRI lesion severity grading and surgically verified lesion grading is scarce and inconclusive. Stress radiography under local anaesthesia and examination under general anaesthesia tendentially agreed with intraoperative findings.

Physical examination is the primary diagnostic tool to evaluate clinically relevant MCL-lesion stability. Different classification systems have been established to objectively assess this severity [4]. The most common system, according to Hughston and the American Medical Association (AMA), uses a three-point scale of medial gapping in 20–30° flexion of the knee [20, 22]. Other classification systems differ with respect to clinical symptoms at different flexion angles and different valgus loads, which are highly examiner-dependent and make reliable comparison difficult [21, 25, 27, 31–33, 42]. Hence, stress radiography, ultrasonography, and magnetic resonance imaging are supposed to help guide further treatment recommendations.

MRI is the most common imaging method for assessing periarticular soft tissue lesions of the knee. In most studies, a static examination protocol was used [31–35, 39–41, 45, 47–50]. Dynamic examinations under specific and unspecific valgus forces are rarely performed in clinical practice but show high reliability for medial gapping [30]. As a result, primarily direct characteristics of MCL lesions were described rather than a dynamic evaluation of the amount of medial gapping under stress. Depending on specific lesion characteristics, interobserver reliability ranged between moderate and almost perfect agreement, which is likely to be improved due to advanced soft tissue resolution and better multiplanar imaging capabilities [33]. In this context, relative to intraoperative findings, MRI showed better performance in grading lesion severity (79–86%) than it did in reliably predicting lesion location (52–75%). This diverging interpretation (MRI vs. surgery) of the same lesion appears to be a major limitation, which is particularly evident in the interpretation of oblique MCL ruptures [39]. Also, MRI grading of MCL lesions is currently performed using various classification systems. The lack of consensual classification due to these numerous grading systems contribute to this diverging interpretation and lead to noncomparable studies: e.g. Schweitzer et al. comparing their grading system with specific clinical symptoms compared to Rasenberg et al. correlating MRI findings according to Petermann et al. [51] with clinical findings using valgus–varus laxity testing [32, 33]. The difficult MRI-morphological differentiation between atraumatic and traumatic MCL abnormalities increases this area of conflict [34]. The most important exception, and with a high degree of consensus, is the Stener-like lesion. The overall accepted characteristic morphological “wave-sign” shows an overall high correlation with clinical findings as defined by a grade III instability and surgical outcome [45, 47]. However, standardized, prospective studies with reliability testing are still needed to verify this relationship.

Compared to static MRI images, ultrasonography is a dynamic tool that can be used to directly detect characteristics of MCL lesions or analyse MCL function parameters such as medial gapping, both with high interobserver reliability [27, 36, 42, 43, 52]. This modality has been utilized with or without defined valgus force and in different flexion angles. A high correlation between MCL-lesion detection in ultrasonography and clinical examination was observed without a grading differentiation [36]. Additionally, in comparison with fluoroscopic measurement methods of medial gapping, ultrasonography also demonstrated no significant differences and high interobserver reliability [52]. Based on this review, ultrasonography seems to be a reliable, radiation free alternative in the detection of medial gapping and could help in objectifying clinical instability of the MCL. Although high reliability with this modality was reported in the included studies, there was a considerable amount of diversity in study designs and measurement methods which led to high variation in the magnitude of medial gapping present between investigations [42, 43, 52]. Relative to MRI, ultrasonography was less sensitive and specific [27]. This supports the idea that MRI findings may overestimate injury severity with respect to clinical lesion stability. However, in all studies, only superficial structures were assessed and there was no specific anatomical differentiation. To date, there is no current consensus on a standard protocol of ultrasonography examination and classification of MCL lesions. Further standardized, high-quality studies utilizing ultrasonography in this capacity are needed.

Stress radiography has regained more relevance in the last decade with respect to MCL-lesion investigation. This modality is widely accessible in clinical practice and demonstrates high reliability in assessment [53]. However, stress radiography retains the limitations of higher radiation dosage relative to the other studied modalities as well as only being able to provide indirect visualization of structures suspicious of injury. In contrast to earlier hypotheses, minor values of medial gapping, even as low as 3 mm, have been associated with complete MCL lesions [20, 26]. However, several study designs, measurement methods, and imaging options have resulted in high variability of measurement. For example, imaging the most medial aspect of the femoral condyle and corresponding tibia, or the most distal aspect of the medial femur and corresponding tibia of the same knee will yield results that are difficult to compare due to different viewing angles [25, 26, 37]. Despite this variability, the trend of increasing medial joint space opening remains highly correlated to the grade of MCL lesion ultimately diagnosed. Furthermore, concomitant lesions of other stabilizing knee ligaments such as the anterior cruciate ligament magnifies the amount of medial joint space opening and clinical instability present (Table 4) [26, 30, 37, 42–44, 52].

Overall, a moderate-to-strong correlation for MCL lesions was observed among imaging and clinical examination findings. In cases of absent correlation, MCL lesions were observed to be overestimated in MRI assessment relative to clinical evaluation. Despite this inconsistency, the overall correlation for detecting MCL lesions between MRI and clinical examination was high. MRI is highly sensitive in detecting abnormalities of the medial aspect of the knee, but standardized, reliable MRI classifications are still needed to differentiate between clinically relevant MCL-lesion signs and subclinical morphological abnormalities.

Data describing the correlation between MRI-based MCL-lesion severity grading and surgically verified MCL-lesion grading are scarce. Additionally, of the evidence that is present, surgical findings are not described in high detail. Due to this shortcoming, indirect arthroscopic findings and direct intraoperative findings have been equally weighted as a reference in the literature. This generalization leads to inconclusive results with high variance. Additionally, stress radiography under local anaesthesia and examination under general anaesthesia agreed tendentially with intraoperative findings. However, within this comparison as well, detailed intraoperative results are sparse and lack detail. Further intraoperative assessment data are needed to strengthen the relationships reported in this review.

This study has several limitations. Studies with relevant information that were not written in English may be missing in our review due to our exclusion criteria. Furthermore, we analysed studies with a focus on imaging modalities. Studies with a focus on postoperative outcomes and anatomy were excluded. As a result, some studies with complementary imaging information could have been missed. In addition, the studies showed a large heterogeneity of findings, making them difficult to compare and unfeasible to perform a meta-analysis. Finally, this systematic literature review can only be as strong as the studies that were included and analysed, therefore, it is limited to the quality of evidence of the source literature. As a result, conclusions made in this review should be interpreted individually with some degree of caution to reflect this limitation.

## Conclusion

Although there is a paucity of high-quality literature reliably comparing different imaging modalities based on validated gradings, MRI has demonstrated high reliability in its ability to detect a MCL lesion but has limitations to predict the exact lesion location and clinical relevance. Overall, as MCL injuries are complex the consideration of their treatment options should be based on the combination of clinical findings, and imaging.
